# Comparison of cell-based assays to quantify treatment effects of anticancer drugs identifies a new application for Bodipy-L-cystine to measure apoptosis

**DOI:** 10.1038/s41598-018-34696-x

**Published:** 2018-11-05

**Authors:** Nita Kumar, Rayhaneh Afjei, Tarik F. Massoud, Ramasamy Paulmurugan

**Affiliations:** 1 Cellular Pathway Imaging Laboratory (CPIL), Molecular Imaging Program at Stanford, Stanford University School of Medicine, 3155 Porter Drive, Palo Alto, CA 94305 USA; 2 Laboratory of Experimental and Molecular Neuroimaging (LEMNI), Molecular Imaging Program at Stanford, Stanford University School of Medicine, 300 Pasteur Drive, Grant S-031, Stanford, CA 94305 USA

## Abstract

Cell-based assays that measure anticancer drug effects are essential for evaluating chemotherapeutic agents. Many assays targeting various cellular mechanisms are available, leading to inconsistent results when using different techniques. We critically compared six common assays, as well as a new assay using Bodipy.FL.L-cystine (BFC), to identify the most accurate and reproducible in measuring anticancer drug effects. We tested three common chemotherapies (methotrexate, paclitaxel, and etoposide) in two cell lines (Ln229 and MDA-MB231). Spectroscopic assays such as Cell Titer Blue, and 2′,7′-dichlorofluorescin diacetate (DCFDA) yielded a strong drug dose response, especially for paclitaxel and etoposide (R^2^ = 0.9). MTT and Calcein-AM fluorescent dye-based assays were less consistent in that regard. Among three flow cytometry assays, Propidium Iodide (PI)-based DNA content analysis and a new BFC-based glutathione-redox (GSH) assay produced drug dose dependent results. Compared to PI, BFC showed a better correlation (R^2^ = 0.7–0.9) in depicting live and apoptotic cells. We found that the combination of Cell Titer Blue spectroscopy and BFC flow cytometry assays were most accurate in assessing anticancer drug effects by clear distinction between live and apoptotic cells, independent of drug mechanism of action. We present a new application of BFC as an agent for measuring cellular apoptosis.

## Introduction

Evaluation of drugs for their potential anticancer effects is essential when determining their specificity in inducing cancer cell apoptosis^1^. A distinction early in this evaluation must be made between apoptotic and necrotic cell death, i.e. desirable anticancer drug-induced programmed cell death and not simple nutrient depletion associated necrosis^2–5^. Use of cancer cell-based assays are therefore a critical step in studying potential mechanisms of actions of chemotherapeutics before they can be pre-clinically validated using animal models and any subsequent clinical evaluation in humans^6^. Cell-based assays are powerful laboratory tools used in the process of drug discovery and during preclinical validation, but, to date, a wide range of assays that target different cellular mechanisms have been used for anticancer drug evaluation in cells^7,8^. Unfortunately, confusion arises on account of the large variations in results obtained from different reports when using the same drug to assess for efficacy of anticancer cell kill. Thus, it is often difficult to experimentally reproduce such results owing to inconsistent use of various assay systems at different times, and widely discrepant conditions used in experiments by different researchers.

The (3-(4,5-dimethylthiazol-2-yl)-2,5-diphenyltetrazolium bromide) tetrazolium reduction (MTT) cell proliferation assay has been widely used and is considered as a gold standard for measuring cell viability and drug cytotoxicity. However, use of MTT has proven inconsistent and nonspecific in many experimental circumstances^2–5,9,10^. Alternative assays using fluorescent or colorimetric dyes such as, Cell Titer Blue (CTB), Propidium Iodide (PI), Calcein AM, 2′,7′-dichlorofluorescin diacetate (DCFDA), and Annexin V labeled with different fluorophores have also been used for measuring anticancer effects of drugs in cells, but with many instances of similarly unreliable results^11–15^.

Bodipy®.FL.L-cystine (BFC) is a marker that fluoresces in the presence of mixed disulphides resulting from the thiol specific exchange with thiolated biomolecules in live cells^16^. Cells under stress can import more L-cystine through an active xCT transporter^17^ to maintain an active non-enzymatic glutathione-based antioxidant defence mechanism and system^18^. Since BFC is a dye labelled L-cystine, it can indicate the amount of stress experienced by a cell owing to therapeutic induction by chemotherapy. However, its role in potentially assessing and quantifying apoptosis in cells has not been considered or studied hitherto. Use of BFC to quantify apoptosis would allow researchers to identify new anticancer drugs with high specificity and sensitivity, with the potential to benefit patients receiving cancer chemotherapy in the future.

Since several advantages and disadvantages have been reported for each assay, here we perform a critical head-to-head comparison of six commonly used cell-based assay systems along with BFC as a potentially new agent to measure cancer cell apoptosis. We aim to identify an assay(s) that independently or in combination can lead to accurate and reproducible measurements of the therapeutic effects of anticancer drugs. We study three drugs: (1) Paclitaxel, a microtubule destabilizing drug that induces mitotic arrest; (2) Methotrexate, an anticancer drug that inhibits the enzyme dihydrofolic acid reductase, which is important for DNA synthesis; and (3) Etoposide, an anticancer drug targeting DNA topoisomerase II and preventing DNA repair and cell growth arrest. We also use two different cell lines, Ln229 (glioblastoma cells with mutant p53 background) and MDA-MB231 (triple negative breast cancer cells with mutant p53 background). We methodically evaluate all possible combinations of these assays, drugs, and cell lines to quantitatively define the extent of viable and apoptotic cells upon treatment. We establish that the combination of a Cell Titer Blue spectroscopic assay and a BFC based flow cytometry assay is accurate and efficient in measuring the anticancer effect of drugs independent of their mechanisms of action. In addition, we present a new application of BFC as an agent for measuring cellular apoptosis.

## Results

### Concept of monitoring xCT cystine/glutamate antiporter activity to measure early apoptosis initiation in cancer therapy using BFC

**Figure 1:**
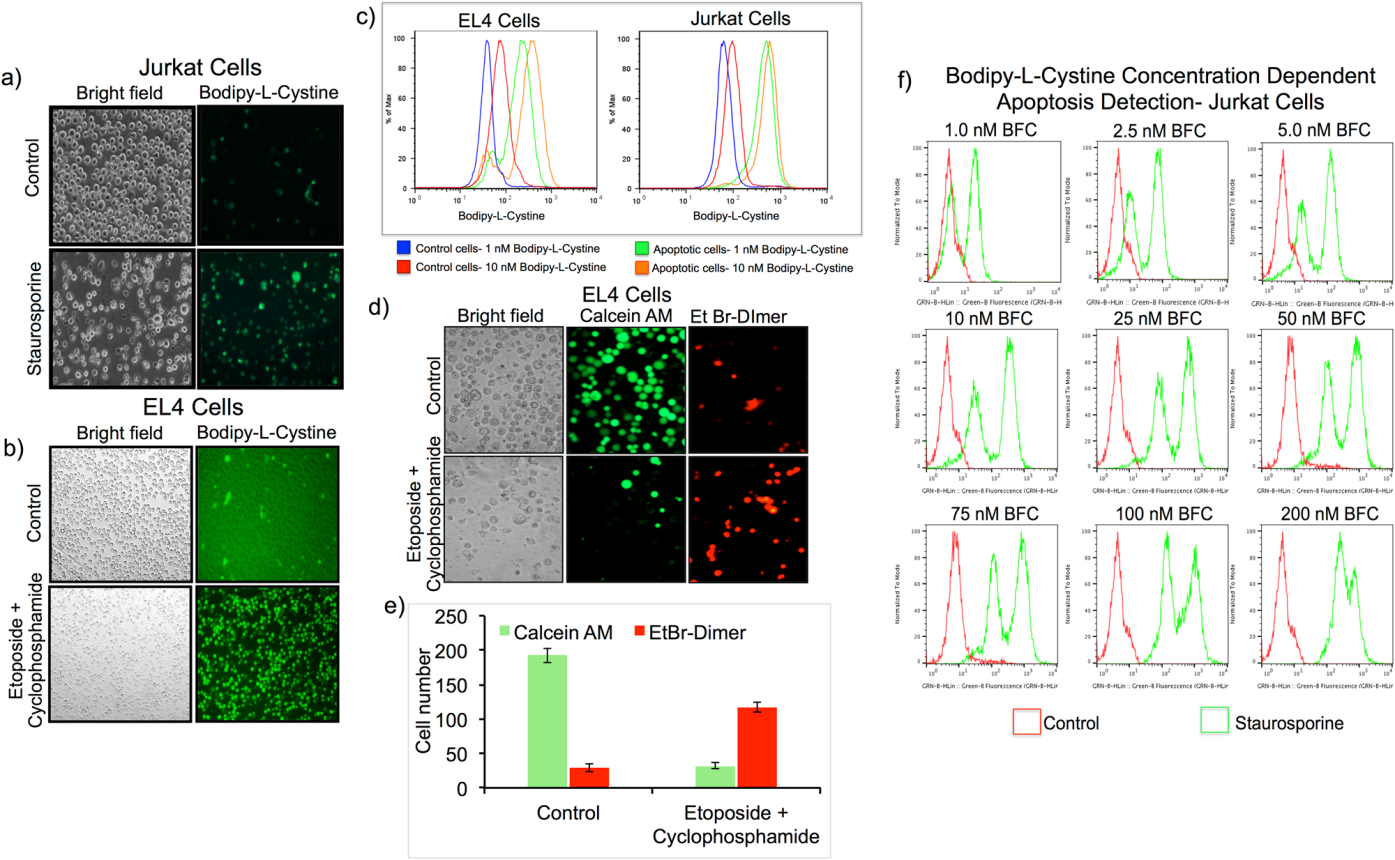
Evaluation of Bodipy-L-Cystine (BFC) as a potential agent for measuring chemotherapeutic drug induced apoptosis in cancer cells. (**a**,**b**) Fluorescent microscopic images of Jurkat cells treated with 0.5 μM of staurosporine (**a**) and EL4 cells treated with a combination of etoposide (50 μM) and cyclophosphamide (10 μg/ml) (**b**) stained with BFC (1 nM) for visualizing apoptotic cells. (**c**) FACS analysis of Jurkat cells treated with 0.5 μM of staurosporine (right) and EL4 cells treated with a combination of etoposide (50 μM) and cyclophosphamide (10 μg/ml) (left) for comparison of BFC concentration (1 nM and 10 nM) dependent apoptosis measurement. (**d**) Fluorescent microscopic images of EL4 cells treated with a combination of etoposide (50 μM) and cyclophosphamide (10 μg/ml), and stained with Calcein AM (green; live cells) and Ethidiumbromide homodimer (red; dead cells) for live and dead cell differentiation. (**e**) A quantitative graph showing the live and dead cell populations from the fluorescent microscopic data shown in figure (**d**). (**f**) FACS analysis of Jurkat cells treated with 0.5 μM of staurosporine and stained with different concentrations of BFC (1 nM to 200 nM) to identify the effective concentration of BFC required for efficient apoptosis measurement.

**Figure 2:**
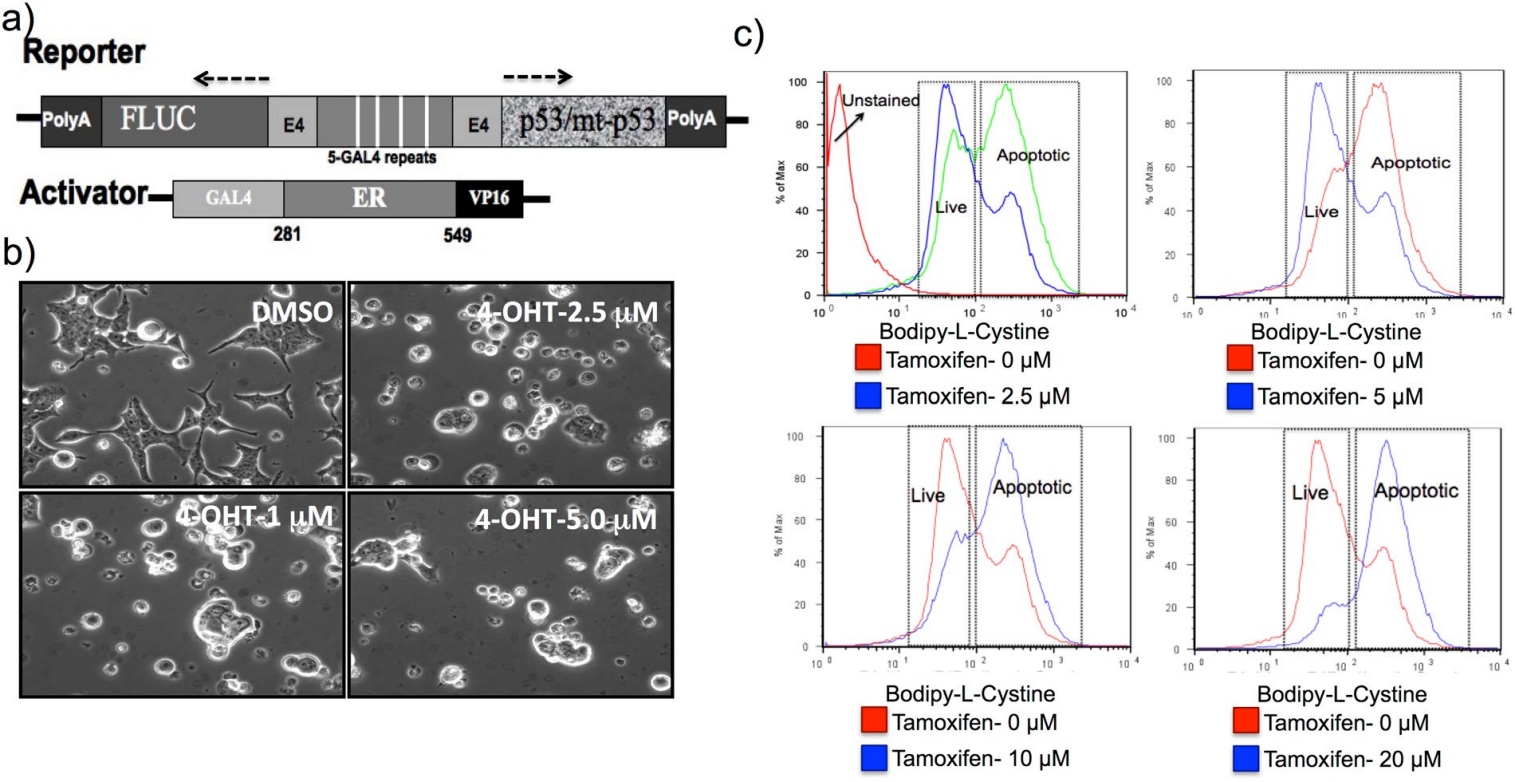
p53-mediated apoptosis induction in HepG2 cells measured by BFC. (**a**) Schematic illustration of a Tamoxifen-regulatable p53-gene expression system to conditionally induce apoptosis in cancer cells. (**b**) Microscopic images of HepG2 cells after apoptosis induction by Tamoxifen-regulated p53 expression. (**c**) FACS analysis of HepG2 cells induced to express different levels of p53 protein by various concentration of Tamoxifen (0–20 μM) and stained apoptotic cells by BFC.

The xCT-cystine/glutamate-antiporter-glutathione (GSH) mechanism is crucial for cell survival in various cellular conditions, including chemotherapy-induced cell stress and apoptosis. We therefore aimed to assess the potential use of L-cystine (a substrate involved in GSH synthesis) as an early marker for apoptosis evaluation after chemotherapy. The xCT-cystine/glutamate antiporter is an important transporter located on cell membranes, assisting the transport of extracellular cystine into cells. Cells require excess cystine to synthesize GSH under stress conditions, including when oxidative stress arises during cancer therapy. GSH, a tri-peptide that serves as an antioxidant, prevents cellular damage caused by reactive oxygen species such as free radicals and peroxides. Cystine transport mainly occurs through the xCT antiporter with the exchange of intracellular glutamate. In this study, we hypothesized that upregulation of the cystine transport through xCT antiporter is an early event that is activated during cancer therapy. Measuring intracellular cystine transport would not only indicate the presence of early stage apoptosis, but can also indicate whether the cancer cells respond to a given therapy during anticancer treatment **(**Suppl. Fig. 1a). BFC can enter cells but it cannot be metabolized. It is trapped inside cells and thus may form the basis of a novel approach to quantitatively evaluate the real-time accumulation of extracellular cystine inside cells. To test our hypothesis, we used chemically (staurosporine and etoposide/cyclophosphamide) induced apoptosis in EL4 and Jurkat cells, and p53 tumor suppressor protein gene therapy mediated enhancement of apoptosis in HepG2 cells (Figs 1 and 2).

Our objective was to identify an assay that can potentially evaluate chemotherapy-induced early apoptosis in cancer cells. We selected BFC as a compound for our evaluation, to take advantage of its fluorescent properties for FACS and fluorescence microscopic analysis. We assessed the uptake of BFC in Jurkat cells treated with 0.5 μg/ml of staurosporine for 6 h and EL4 cells treated with a combination of etoposide (50 μM) and cyclophosphamide (10 μg/ml) for 20 h. We found significant uptake of BFC by apoptotic cells when compared to control cells as analysed by fluorescent microscopy and FACS (Supp. Fig. 1a and Fig. 1a–c). To further confirm the uptake of BFC occurring via the xCT-cystine/glutamate antiporter, we used sulfasalazine, a glutathione analogue that inhibits this antiporter. We showed that, with BFC, the Jurkat cells treated with staurosporine (a drug that normally causes cell death through apoptosis) underwent apoptosis at different levels, resulting in three distinct fluorescent peaks indicative of early, intermediate and late stage apoptosis (Supp. Fig. 2). The middle peak, B, represented cells in an early/intermediate state of apoptosis. Since these early apoptotic cells were functionally active, the prevention of further effects of staurosporine (by glutamate/cystine antiporter-mediated sulfasalzine treatment) was able to prevent the cells from moving into a late apoptotic stage. Hence, the result of this experiment clearly confirmed that BFC was able to measure apoptosis, and not the dead cells.

We co-incubated cells treated with staurosporine with 1 nM of BFC and 0.15 mM of sulfasalazine for 30 min at 37 °C and analysed by FACS. We observed significant reduction in the fluorescent signal from apoptotic cells when co-treated with sulfasalazine compared to staurosporine alone (Supp. Fig. 2a). We also tested the consistency of this property in another cell line, MDA MB231, by treating with 0.05, 0.1 and 0.2 mM sulfasalazine under conditions similar to those for Jurkat cells. The results showed a sulfasalazine dose-dependent inhibition of BFC uptake (Supp. Fig. 2b).

### Optimization of BFC concentration to distinguish chemotherapy-induced apoptosis in cancer cells

To identify the optimal concentration of BFC for efficient detection of chemotherapy-induced apoptosis, Jurkat cells treated with 0.5 μg/ml staurosporine for 6 h or EL4 cells treated with a combination of etoposide (50 μM) and cyclophosphamide (10 μg/ml) for 20 h were stained either with 1 nM or 10 nM of BFC for 30 min. We analyzed the cells using FACS, and found a similar level of specificity and sensitivity for both concentrations in measuring apoptotic cells (Fig. 1c). However, the control cells stained with 1 nM BFC revealed minimal background signal compared to using 10 nM BFC. Hence, we used 1 nM BFC for all subsequent experiments.

Additionally, we used an independent dead and live cell staining kit from Molecular Probes to confirm these results of testing BFC. We stained EL4 cells treated for 20 h with etoposide and cyclophosphamide combined with Calcein AM, an esterase activatable fluorescent probe that detects live cells only, and cell impermeable ethidium bromide (EtBr) homodimer as a DNA staining agent that stains only the cells possessing a destabilised membrane potential normally found in apoptotic cells. The results clearly confirmed that BFC uptake matched with Calcein AM and the EtBr homodimer-mediated live and dead cell assay (Fig. 1d,e). To further evaluate the effective concentration of BFC required for efficient apoptotic detection without much non-specific uptake or toxicity to control cells, we performed a BFC dose study spanning a range of 1 nM to 200 nM in Jurkat cells treated with 200 nM of staurosporine for 6 h. The results showed that concentrations of up to 10 nM were able to detect both live and apoptotic cell populations with equal sensitivity, without affecting the viability. But above 10 nM we found significantly higher uptake of BFC by control cells, which affected the ratio of live to dead cells. Interestingly, 1 nM showed better results without much non-specific uptake by live cells **(**Fig. 1f**)**. We therefore used 1 nM in the rest of our study.

### Detection of p53-mediated apoptosis in HepG2 cells evaluated using BFC

Although to this point we had evaluated measuring anticancer drug-induced apoptosis using BFC in Jurkat and EL4 cells, we further reflected on whether xCT-antiporter-based cystine uptake is a more general phenomenon occurring also after other mechanisms that induce cellular apoptosis, as might follow gene therapy, for example. Therefore, to systematically measure p53 dose-dependent apoptosis induction, we used a Tamoxifen-regulated conditional gene expression system (Fig. 2a). We used a co-expressed Firefly luciferase (FLUC) reporter gene to indirectly monitor the expression of p53. We induced HepG2 cells stably expressing the conditional p53 system with various concentrations of 4-hydroxy Tamoxifen (2.5, 5, 10 and 20 μM), stained with BFC, and then analysed cells using FACS. We observed a Tamoxifen concentration-dependent increase in cell death by microscopic imaging **(**Fig. 2b**)** and FACS analysis **(**Fig. 2c**)**.

### Comparison of various assays for their predictive suitability in measuring chemotherapy-induced apoptosis and their comparison with BFC

#### Flow cytometry assays

**Figure 3:**
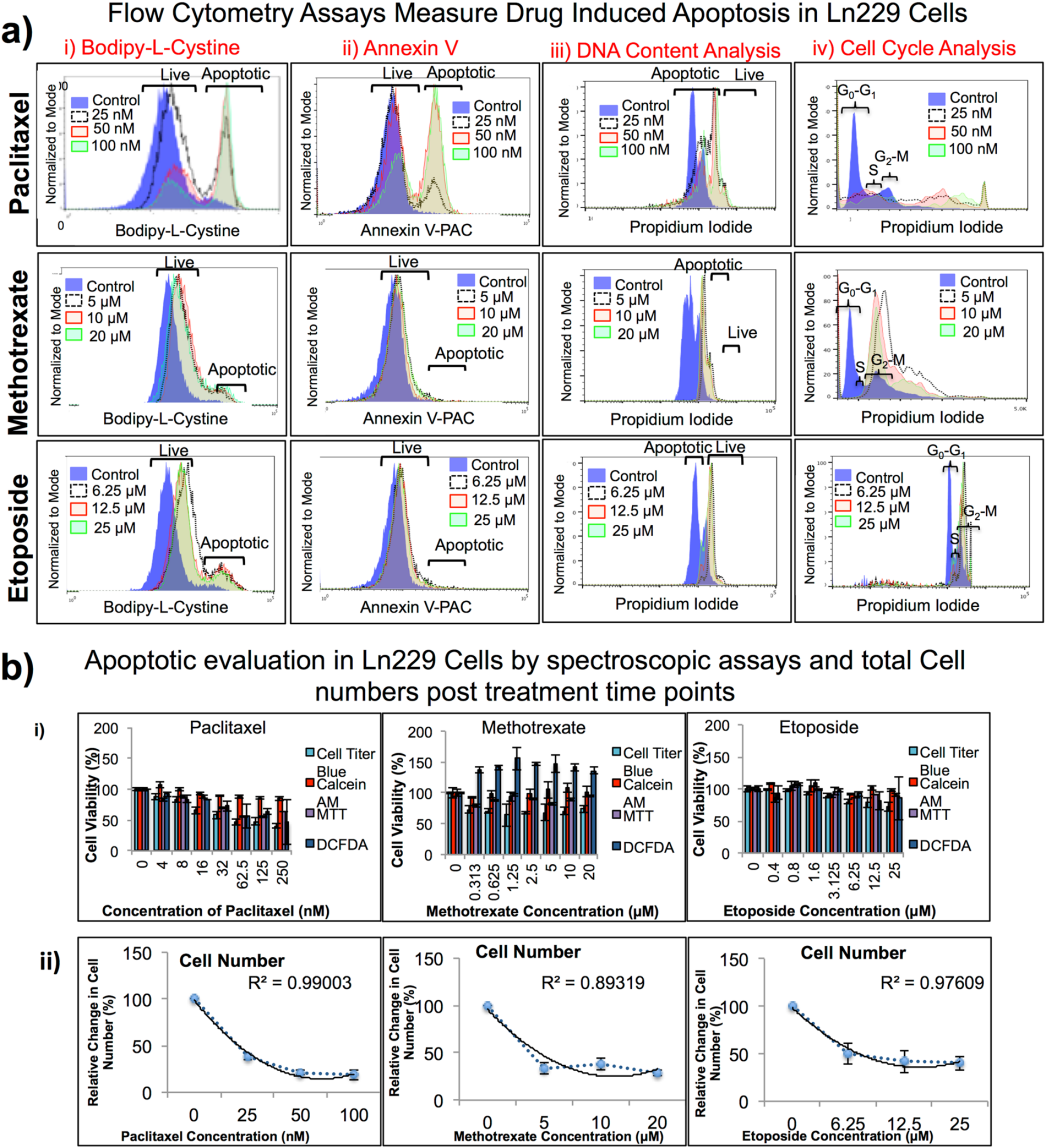
(**a**) Flow cytometry assays measure drug-induced apoptosis in Ln229 cells. Comparison of anticancer drugs in Ln229 GBM cells evaluated using three different common FACS assays (PI staining based DNA content analysis, Annexin V based staining, and Bodipy-L-Cystine based apoptosis staining) to cover cell viability, cell proliferation, cellular stress, apoptotic and live cell populations, and total number of cells present after 48 h post drug treatment. The results show that not all assays measure the cell viability at equal sensitivity. (**b**) Spectroscopy assays measure drug-induced apoptosis in Ln229 cells. We used four different spectroscopy assays (MTT, Calcein AM, Cell Titer Blue, and DCFDA). The results here also show that not all assays measure the cell viability at equal sensitivity. The Cell Titer Blue assay was most consistent when compared to other spectroscopic assays (i). We also measured total cell numbers 48 h after drug treatment (ii).

Our aim was to determine the optimal assay(s) to accurately measure the therapeutic effects of anticancer drugs while evaluating the drug mechanism of action. We used paclitaxel at 0, 25, 50 and 100 nM, methotrexate at 0, 5, 10, and 20 μM, and etoposide at 0, 6.25, 12.5, and 25 μM concentrations in both MDA-MB231 and Ln229 cells; and compared treatment results using three different flow cytometry assays (FACS). Among the three assays, Propidium Iodide (PI) based DNA content analysis, and BFC based GSH assay produced results that display the dose dependent effect for both cell lines **(**Fig. 3a, Suppl. Fig. 3a, Tables 1 and 3**)**. BFC showed a better correlation (R^2^ = 0.7–0.9) in accurately depicting live versus apoptotic cell populations, when compared to PI. Annexin V staining showed only a slight change in live versus apoptotic cell populations in comparison to BFC and PI. (It) Annexin V was not consistent in accurately determining live versus apoptotic populations for the various drug treatment conditions.

#### Spectroscopic assays

**Figure 4:**
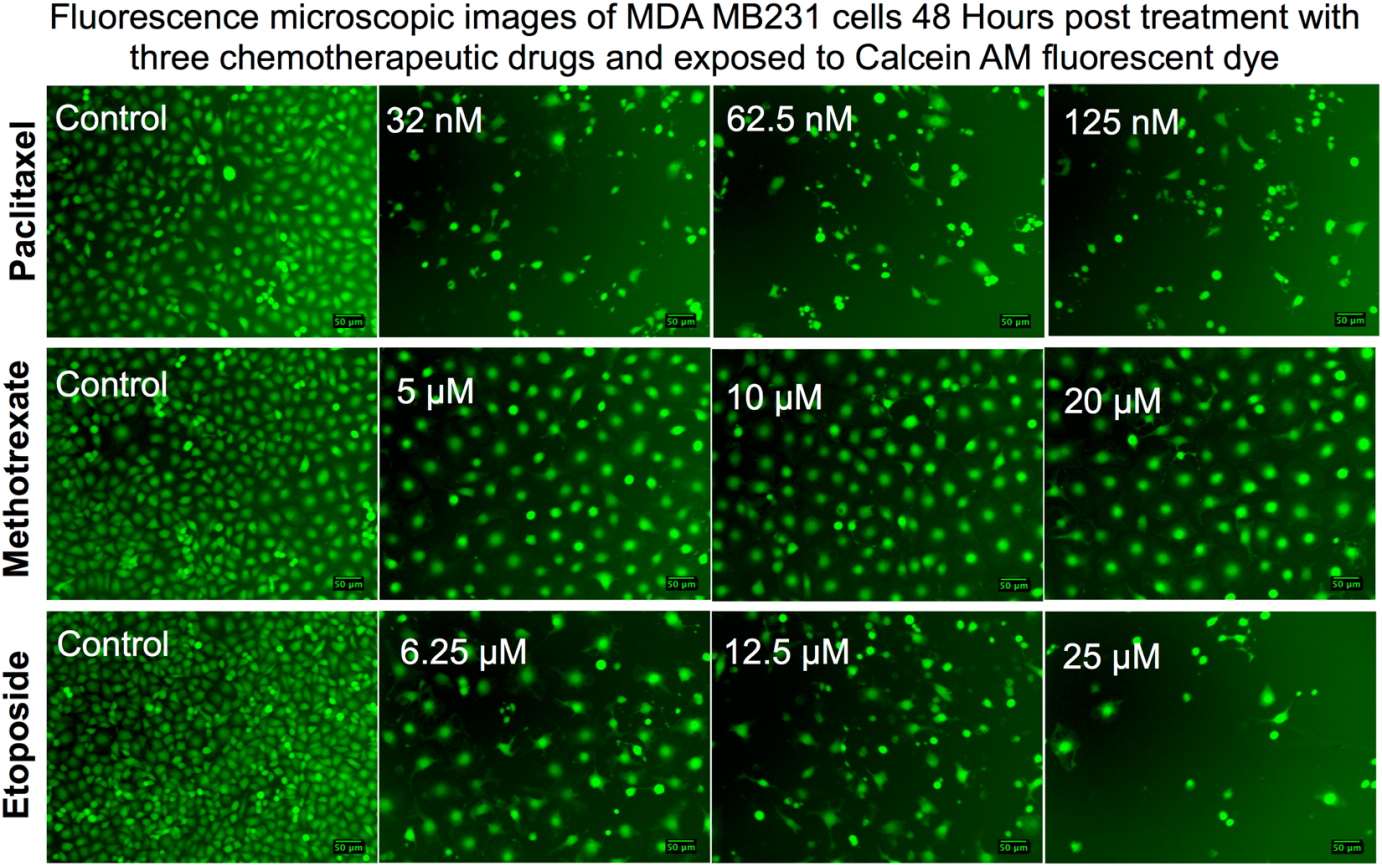
Fluorescence microscopic images of MDA-MB231 cells 48 h post treatment with three different chemotherapeutic drugs and stained with Calcein AM fluorescent dye. Fluorescent microscopy images of MDA-MB231 cells stained with Calcein AM (green; live cells) for live and dead cell differentiation after apoptosis induction with paclitaxel (top), methotrexate (middle), and etoposide (bottom) after 48 h of drug treatment.

Alongside flow cytometry assays, we also assessed similar concentrations of drugs in both Ln229 and MDA-MB231 cells using different spectroscopic assays (Cell Titer Blue, MTT, Calcein AM, and DCFDA). Among the four spectroscopic assays, Cell Titer Blue and DCFDA showed high correlation for drug dose response in both cell lines, especially for cells treated with paclitaxel and etoposide (R^2^ = 0.9) **(**Fig. 3b, Suppl. Fig. 3b**)**. MTT assay and Calcein AM fluorescent dye -based assays were not consistent and did not show any dose dependent effect (R^2^ = 0.2–0.4). We also used the fluorescent property of Calcein AM for microscopic analysis. The images clearly showed the dose dependent effect in MDA-MB231 cells upon treatment with each drug **(**Fig. 4**)**. Similarly, we observed a decrease in cell numbers as the concentration of the drug increased in Ln229 cells as well **(**Suppl. Fig. 4**)**. These results were consistent in both cell lines and for all three drugs. We were also able to confirm the effect of the drugs on both cell lines by observing the cell morphology on brightfield microscopic images for Ln229 and MDA-MB231 cells **(**Suppl. Figs 5 and 6**)**.

### Time-point analysis in MDA-MB231 cells defines a combination of two assays that can predict cell viability and mechanism of action with high accuracy

**Figure 5:**
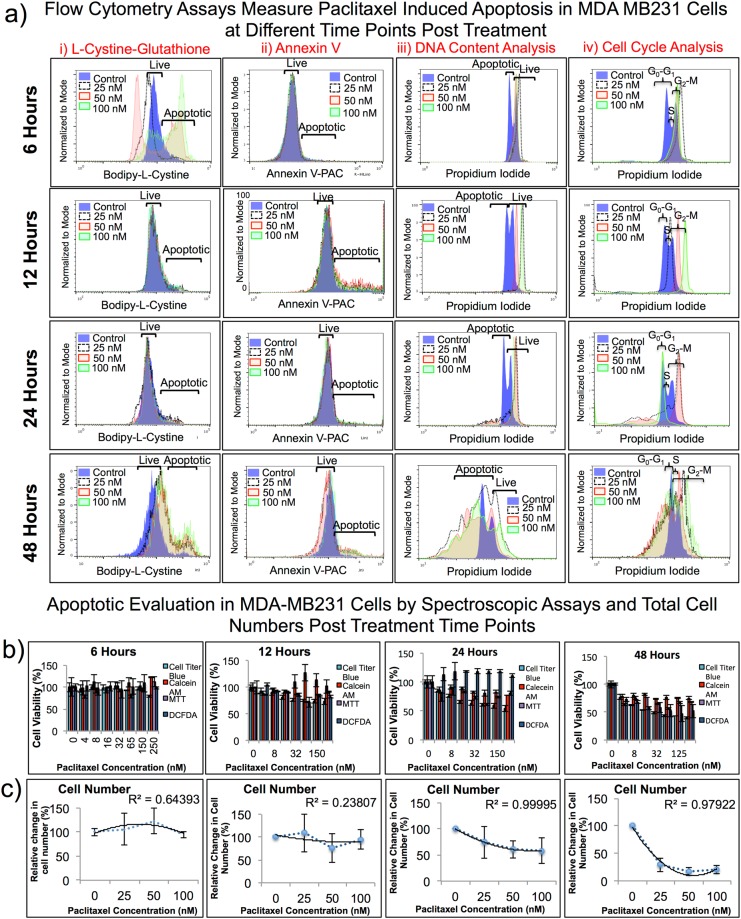
Paclitaxel dose- and time-dependent apoptotic measurement in MDA-MB231 cells treated with three different drugs doses, for four different durations, and assessed by seven different assays. (**a**) Flow cytometry assays measure apoptotic effect in MDA-MB231 cells treated with paclitaxel at 25, 50, and 100 nM concentrations analysed at 6, 12, 24, and 48 h post treatment. (**b**) Spectroscopic assays measure apoptotic effect in MDA-MB231 cells treated with paclitaxel at 25, 50, and 100 nM concentrations analysed at 6, 12, 24, and 48 h post treatment. Cell Titer Blue has the highest R^2^ value which indicates that the assay performs well with maximum consistency. BFC displays two distinct populations, as the labels above show, that can be easily differentiated when determining viable and apoptotic cells. (**c**) Cell number decreases as the concentration of anticancer drugs increases.

We extended our drug dose dependent study to measure the accuracy of therapy predictability at various time-points after drug treatment in MDA-MB231 cells. We used similar doses for all three drugs (paclitaxel, methotrexate and etoposide). After initial drug exposure, we allowed the cells to grow for 6, 12, 24 and 48 h in the presence of the drugs and then used these cells for different spectroscopic assays and FACS analysis, as detailed previously. The Cell Titer Blue assay showed the highest coefficient of determination value (R^2^ = 0.9) in all time-point evaluations, which indicated that the assay performed consistently well **(**Fig. 5a, Suppl. Table 2**)**. BFC displayed two clear cell populations that were easily differentiated as live and apoptotic **(**Fig. 5b**)**. Annexin V and PI did not show as clear a distinction for treatment effects as we observed when using BFC.

Finally, to confirm the correlation between BFC uptake and apoptosis, we treated Jurkat cells with different concentrations of staurosporine (0, 50, 100 and 200 nM) for 6 h and evaluated for apoptotic cell population using BFC uptake, and for caspase activity using the Promega Luciferase-Luciferin based DEVD cleavage assay, which measures Caspase 3/7 activity. The results showed significant correlation between these two results (Supp. Fig. 7).

## Discussion

Anticancer drug development remains a continuous process in an attempt to identify new or repurposed drugs, those that might be more effective in treating resistant cancers, or those that could show better treatment effects in combination with other drugs^13,19,20^. Hence, the development of cell-based assays for screening new drugs, and therapeutic evaluation of drugs for their potential treatment effects and toxicity is evolving continuously^21–23^. There are numerous flaws and limitations when using currently available cell-based assays. In a commonly used assay, MTT is converted into a water insoluble purple formazan crystal in live cells by mitochondrial dehydrogenase enzyme. However, this compound interferes with several other cellular mechanisms, leading to inconsistent results^14^. This has been reported in prior studies where drugs such as Imatinib, rottlerin, ursolic acid, verapamil, resveratrol, genistein, and some polypeptides interfere with the MTT assay^5^. Similarly, treatment of U251, T98G and C6 glioblastoma cells with temsirolimus (an inhibitor of mTOR kinase), U0126 (an inhibitor of MEK1/2 kinases), and temozolomide (a DNA alkylating agent) have shown unpredictable results when the MTT assay was used for measuring cytotoxicity^3^. Treatment of MDA-MB-231, MCF-7 and MCF-12A cell lines with 3-bromopyruvate, lonidamine, and 2-deoxyglucose, also produced inconsistent results and low assay reproducibility when MTT assay was used for evaluation^4^.

Alternatively, assay methods using fluorescent or colorimetric dyes such as Cell Titer Blue, Propidium Iodide (PI), Calcein AM, DCFDA, and fluorescently labelled Annexin V peptide have been used for measuring anticancer effects of drugs in cells^13,14^. Calcein AM allows the discrimination between outer and inner membrane (IM) permeabilization after drug treatment. However, it cannot identify transient or reversible IM changes that may occur during cell death owing to some unrelated molecular mechanisms. It also detects the loss of barrier function of the IM to ions (in particular to Co^2+^)^11^. H_2_DCFDA is a small molecule dye that passively diffuses into cells where it is converted to non-fluorescent DCFH by intracellular esterases, which is further oxidized to highly fluorescent 2′,7′-dichlorofluorescein (DCF) by intracellular reactive oxygen species (ROS). Hence, H_2_DCFDA serves to indirectly measure intracellular ROS levels in cells. However, in some instances of chemotherapy-induced cell death, the temporary ROS overload alone does not always result in cell death. Probes specific for an individual ROS may show partial cross-reactivity to overgeneration of ROS, which is very often a prelude to mitochondrial permeability transition^11^. In addition, exclusion dyes are extruded by healthy cells, yet are taken up by cells with ruptured plasma membranes^11^.

Propidium Iodide (PI) is a nuclear dye that binds to double stranded DNA by intercalating through base pairs in cells that are permeabilized by fixatives. Measuring the localized high intensity PI-fluorescence is the indicator of live cells, while diffuse low intensity fluorescence measured from fragmented DNA serves as an apoptosis indicator^12^. PI-based FACS analysis indirectly measures the cellular DNA content and its distribution. PI staining shows some advantage in measuring both cell cycle distribution and apoptosis. In contrast, this assay cannot distinguish between apoptotic and necrotic cells, which is important for distinguishing drug-induced programmed cell death from other random events. On the other hand, Annexin V is a peptide that binds to phosphatidylserine (PS), and has been used for measuring apoptosis. PS is a lipid normally located at the inner lining of the plasma membrane in normal cells; it translocates to the cytoplasmic surface of the outer leaflet in apoptotic cells, where it can be detected using fluorescently labelled Annexin V peptide using FACS analysis^15^. Annexin V staining does not require the fixation of cells for the assay and is specific in distinguishing an early event in the executioner phase of apoptosis. However, Annexin V can also produce non-specific signal by binding to both inner and outer membrane PS peptides when cells have ruptured plasma membranes^11^. Furthermore, PS exposure may be impaired in autophagy-deficient cells.

In addition to studying various existing assays, in this study we identified a fluorescent L-cystine molecule (BFC, which was previously used as a laboratory reagent for labelling proteins and nucleic acids, and for measuring intracellular Trx/GSH levels^24,25^), as a new substrate to measure chemotherapy-induced apoptosis in cells. We optimized the BFC concentration and assay conditions required to accurately measure therapeutic effects under various treatment conditions. These included cells treated with chemotherapeutic drugs, and cells over expressing the p53 tumour suppressor protein as part of an apoptosis-inducing gene therapy system. We tested this assay in three different cell lines where either cells were in treatment with anticancer drugs (Jurkat and EL4 cells) or in cells induced with overexpressed p53 (HepG2 cells). After optimisation, we tested BFC as a new assay agent for comparison with other six commonly used assays for evaluation of anticancer effects.

As such, we evaluated seven different assay systems that work through different mechanisms, and systematically compared their different readouts under similar conditions (Suppl. Fig. 1). We also tested all assays for drug-induced apoptosis measurements in two different cell lines possessing different genetic backgrounds, using different doses of drugs, and at different time points^26–28^. The results of BFC tested in MDA-MB231 and Ln229 cells in response to paclitaxel, methotrexate, and etoposide showed significant match with the results we obtained during its evaluation in Jurkat and EL4 cells. This further confirms the generalizability of the new assay using BFC as a most effective one (amongst the assays compared in this study) for determining chemotherapeutic drug-induced cell viability in different cell lines. Since a GSH-mediated cytoprotective mechanism has been considered an early event in cells responding to physical and chemical stresses, including that following exposure to chemotherapy, the targeting of this particular process using BFC likely explains its relative success when using it as a readout for cell apoptosis, as reflected in the results of this study.

Most currently available cell-based assays have readouts at a standard time-point of 48 h to 72 h after drug treatment. Because of this extended incubation time most of the cells that are responding at early times after treatment are lost at the time the assay is performed owing to disintegration of cells into particulate debris; this alters the ratio between the live and dead cell populations present at the time of analysis. Hence, to identify an ideal time-point needed for performing assay readout to report meaningful results in response to drug treatment, we performed time-point experiments using all the assays. In addition to a conventional single late time-point experiment, we also included early time-points (at 6 h, 12 h, and 24 h) for evaluation; these resulted in more accurate measurements of the distribution of both live and apoptotic populations when comparing the same cells treated with similar drugs and evaluated at later time-points (48 h to 72 h post-treatment). The Cell Titer Blue assay demonstrated the highest coefficient of determination value with more reproducible results compared to other spectroscopy assays. BFC was a better assay for measuring the early response of cells to treatment. Early time-point assessment at 12 h to 24 h is necessary to clearly determine whether a drug induces cytotoxicity, or it simply arrests cell growth and generates a cytostatic effect. Our results also show that there is significant number of cells that respond early to treatment through apoptosis (Fig. 5).

In conclusion, we identified BFC as an optimal agent for measuring anticancer drug-induced cytotoxic effects in cells. We optimized the concentration and the assay conditions needed for optimal measurement of treatment effects of drugs in cells when using this assay. Further, we successfully compared the BFC based flow cytometry assay with six other commonly used assays for evaluating the treatment effects of several drugs in separate cell lines of different genetic backgrounds, and found that spectroscopic assays such as Cell Titer Blue and DCFDA are efficient for measuring drug-induced cell growth arrest. MTT and Calcein AM based spectroscopic assays were not reliable and did not show consistent dose dependent effect. We also identified PI based DNA content analysis and BFC based glutathione assay as effective flow cytometry assays for accurate monitoring of anticancer drug-induced apoptosis in different cancer cells. Taken together, a combination of Cell Titer Blue assay (spectroscopy) and BFC assay (flow cytometry) can be efficient and accurate when assessing the anticancer effects of drugs in cancer cells, by determining cell viability as well as by clearly measuring live and apoptotic cells present after treatment, independent of the mechanism of drug action and the conditions used for analysis. Overall, we present a new application of BFC as an agent for measuring cellular apoptosis.

## Methods

### Chemicals, enzymes, and reagents

We purchased Jurkat human T-lymphoblast cells, EL4 mouse T-lymphoblast cells, HepG2 human hepatocellular carcinoma cells, MDA-MB231 human breast cancer cells, and Ln229 human glioblastoma cells from ATCC (Manassas, VA). We also purchased the following: Bodipy®.FL.L-cystine (BFC) from Life technologies (Grand Island, NY); Propidium Iodide (PI) from Sigma-Aldrich (Milwaukee, WI); Alexa Fluor Annexin V from BioLegend (San Diego, CA); Cell Titer Blue (CTB) from Promega Corporation (Madison, WI); MTT from Life Technologies (Eugene, OR); DCFDA from Sigma-Aldrich (Milwaukee, WI); Calcein AM from Life Technologies (Eugene, OR); etoposide, paclitaxel and hydroxyTamoxifen from Sigma-Aldrich (St. Louis, MO); methotrexate from Tocris Bioscience (Bristol, UK); DMSO from Fisher Scientific (Santa Clara, CA); and cell culture medium, FBS, penicillin, streptomycin, sodium bicarbonate, and all cell culture plates from GIBCO BRL (Frederick, MD). Plasmid vector expressing p53 protein under a Tamoxifen regulatable gene expression system was constructed by standard gene cloning procedures in our laboratory^29^.

### Cell culture

We grew Jurkat human lymphoblast cells as a suspension culture in RPMI medium supplemented with 10% fetal bovine serum and 1% penicillin/streptomycin solution. The cells were replenished once every three days with a cell concentration 2 × 10^5^ to 1 × 10^6^/ml. Similarly, we grew EL4 mouse lymphoblast cells as a suspension culture in DMEM supplemented with 10% horse serum and 1% penicillin/streptomycin solution. U87-MG, HepG2, Ln229 and MDA-MB231 cells were cultured with a high-glucose DMEM medium supplemented with 10% FBS and 1% penicillin/streptomycin solution. We grew the ligand-regulatable HepG2-p53 cells in DMEM with 10% charcoal-treated foetal bovine serum with 1% penicillin and streptomycin. Stable cell lines were cultured with respective antibiotics throughout the period of maintenance.

### Cell transfection and p53 expression

Cell transfections were carried out with Lipofectamine 2000 transfection reagent (Invitrogen, Carlsbad, CA). We used HepG2 cells in a 10 cm culture plate at 80% confluence, plated 24 h in advance, for transfection and stable selection for the expression of p53 protein under a Tamoxifen regulatable system. In brief, we co-transfected cells with 2.5 µg each UAS-TATA-p53 and CMV-GAL4-ER-LBDF-VP16 by using a Lipofectamine 2000 transfection reagent, in accordance with the manufacturer’s instructions. We double selected stable cells by using puromycin (1 μg/ml) and neomycin (25 μg/ml) as selection markers. For transfection, 5 µg of each DNA samples and 15 µl of Lipofectamine 2000 were used in 10 cm culture dishes. We incubated transfected cells for 24 h at 37 °C with 5% CO_2_ and subsequently used these for cell-based experiments.

### Apoptosis induction

For apoptosis induction in EL4 and Jurkat cells, we used exponentially growing cells of 0.5 × 10^6^ cells/ml suspension sub-cultured 24 h before experiment. We collected the cells on the day of treatment, suspended them at 1 × 10^5^ cells/ml, and then co-exposed EL4 cells to etoposide (50 μM) and cyclophosphamide (10 μg/ml) for 20 h, and Jurkat cells to staurosporine (0.5 to 1 μg/ml or 50–500 nM) for 6 h. HepG2 cells stably expressing p53 protein under a Tamoxifen inducible system were plated at 80% confluence 24 h before inducing them with different concentrations of Tamoxifen (0, 2.5, 5.0 and 10 μM) for a further 20 h.

We plated Ln229 and MDA-MB231 cells at 80% confluence in a 6-well plate for 24 h before inducing them with different concentrations of paclitaxel (0, 25, 50, and 100 nM), methotrexate (0, 5, 10, and 20 µM), and etoposide (0, 6.25, 12.5, and 25 µM). We plated Ln229 and MDA-MB231 cells at 80% confluence in a 96-well plate for 24 h before inducing them with different concentrations of paclitaxel (0–250 nM with a 0.5 dilution factor), methotrexate (0–20 µM with a 0.5 dilution factor), and etoposide (0–25 µM with a 0.5 dilution factor).

### BFC staining and FACS analysis

We dissolved BFC in DMSO as a 10 mM stock solution. The stock solution was aliquoted and stored at −80 °C until use. A working solution of 1 µM in HBSS (pH 7.4) was used for staining cells for FACS and fluorescent microscopic analysis. We collected the control cells and cells treated with respective drugs described above (Jurkat and EL4) after respective treatment periods and washed them once with PBS containing 1% FBS. We re-suspended the cells in PBS containing 1% FBS to a concentration of 1 × 10^6^ cells/ml. We stained the cells with 1 nM BFC for 30 min at room temperature, washed once with PBS, and analysed them by FACS-Caliber at FL1-Channel. We analysed 5–10 × 10^3^ cells for each sample. Similarly, we collected the adherent HepG2 and HepG2-p53 cells after treatment with different concentrations of Tamoxifen by treating them with a TripLE cell-disrupting medium. We washed the cells once with PBS, re-suspended, stained, and FACS analysed, as previously described for Jurkat and EL4 cells.

For FACS analysis, we plated both Ln229 and MDA-MB231 cells in 6-well plates at 2 × 10^5^ cells/well in DMEM medium supplemented with 10% FBS. We grew cells overnight and changed to DMEM medium supplemented with 10% FBS and exposed them to the required conditions (e.g. various drugs at different concentrations), each condition being represented by three wells; unexposed cells served as controls in every experiment. Cells were then allowed to grow for 6, 12, 24 and 48 h post exposure to drugs before collecting them for FACS analysis. We collected all cells by trypsinization and suspended them in 0.5 ml of ice -cold PBS. Briefly, cells were spun, washed once in PBS, and suspended in 0.5 ml PBS containing 1 μl BFC. After 30 min of incubation at room temperature, we washed the cells once with PBS, re-suspended in 0.3 ml PBS, and used for FACS analysis. Cells were FACS analysed using a Guava-FACS analyser [EMD Millipore], and we analysed the generated data using FlowJo 8.6.6 Software [Tree Star].

### Cell Viability Assays

#### MTT Assay

The MTT assay is a colorimetric assay for assessing cell viability. Viable cells reduce the yellow tetrazolium dye MTT [3-(4,5-dimethylthiazol-2-yl)-2,5-diphenyltetrazolium bromide] to its insoluble formazan crystals, which are purple in colour. We used this assay to evaluate the cell proliferation and cytotoxicity of paclitaxel, methotrexate, and etoposide in Ln229 and MDA-MB231 cells. We seeded Ln229 and MDA-MB231 cells in a 96-well plate at 5 × 10^3^ cells/well and incubated these for 24 h in 37 °C with 5% CO_2_. We treated the cells with different drugs in various concentrations and incubated the cells for a further 6, 12, 24 and 48 h. For paclitaxel, we used concentrations ranging from 0 to 250 nM. For methotrexate, we used concentrations ranging from 0 to 20 μM. For etoposide, we used concentrations ranging from 0 to 25 µM. After designated durations of drug exposure, we aspirated the cell culture medium and incubated the cells with 50 μl of medium (containing 12 mM MTT) for 2 h at 37 °C in 5% CO_2_. We then carefully removed the medium and dissolved the reduced formazan crystals in 50 μl DMSO by incubating at 37 °C for 30 min in the dark. We calculated cell viability by measuring the absorbance at 540 nm using a microplate reader (Infinite® M1000 Pro, Tecan Group, Switzerland) and comparing them with the control cells.

#### Cell Titer Blue staining and spectroscopic analysis

The Cell Titer Blue is an assay that is based on the ability of cells to convert a redox dye, resazurin, into a fluorescent end product, resofurin^30^. We used this assay to evaluate the cell proliferation and cytotoxicity of paclitaxel, methotrexate, and etoposide in Ln229 and MDA-MB231 cells. We seeded Ln229 and MDA-MB231 cells in a 96-well plate at 5 × 10^3^ cells/well density and incubated these for 24 h in 37 °C within 5% CO_2_. We further incubated the cells for 6, 12, 24 and 48 h after treatment with different concentrations of respective drugs. For paclitaxel, methotrexate, and etoposide, we used concentrations similar to those used for the MTT assay. After designated periods of drug exposure, we aspirated the cell culture medium and incubated the cells with 50 μl of medium containing 25 μl Cell Titer Blue for 2 h at 37 °C in 5% CO_2_. We calculated cell viability by measuring the fluorescence at 560_ex_/590_em_ nm using a microplate reader (Infinite® M1000 Pro, Tecan Group, Switzerland) and comparing the cell viability with the control cells.

#### DCFDA staining and spectroscopic analysis

The DCFDA assay is an assay that measures the reactive oxygen species (ROS) within a cell. Once the dye diffuses into the cell, it is deacetylated by intracellular esterases to a non-fluorescent compound (H_2_DCFDA-AM), which is then oxidized by cellular ROS into a highly fluorescent compound (2′-7′dichlorofluorescein (DCF)). We used this assay to evaluate the cell proliferation and cytotoxicity of paclitaxel, methotrexate and etoposide in Ln229 and MDA-MB231 cells. The cell seeding, treatment durations, and drug concentrations were similar to previously described assays. After designated durations of drug exposure, we aspirated the cell culture medium and incubated the cells with 50 μl of medium containing 1 μM DCFDA for 2 h at 37 °C in 5% CO_2_. We calculated cell viability by measuring the absorbance at 495/529 nm using a microplate reader (Infinite® M1000 Pro, Tecan Group, Switzerland) and comparing the results with the control cells.

#### Calcein AM staining and spectroscopic analysis

The Calcein AM assay is a non-fluorescent dye that can readily enter into live cells. Once inside, the AM (acetomethoxy) is cleaved by esterases and the fluorescent calcein protein remains inside the cells. We used this assay to evaluate cell proliferation and cytotoxicity of paclitaxel, methotrexate, and etoposide in Ln229 and MDA-MB231 cells. The cell seeding, treatment durations, and drug concentrations were similar to previously described assays. After designated durations of drug exposure, we aspirated the cell culture medium and incubated the cells with 50 μl of medium containing 1 μM Calcein AM for 2 h at 37 °C in 5% CO_2_. We calculated cell viability by measuring the absorbance at 495/516 nm using a microplate reader (Infinite® M1000 Pro, Tecan Group, Switzerland) and comparing with the control cells.

#### Annexin V staining and FACS analysis

Annexin V peptide was reconstituted in dilution buffer. The stock solution was aliquoted and stored at 4 °C until use. For FACS analysis, we plated both Ln229 and MDA-MB231 in 6-well plates at 2 × 10^5^ cells per well in DMEM medium supplemented with 10% FBS. We grew cells overnight and changed to DMEM medium supplemented with 10% FBS and exposed them to the required conditions (e.g. various drugs at different concentrations), each condition being represented by three wells; unexposed cells served as controls in every experiment. Cells were then allowed to grow for 6, 12, 24 and 48 h after drug exposure. We collected all cells by trypsinization and re-suspended them in 0.5 ml ice cold PBS. Briefly, cells were centrifuged, washed once in PBS, and re-suspended in 100 μl binding buffer containing 2 μl of Annexin V. After 15 min, we added 1 ml PBS to the samples. The samples were then centrifuged at 5,000 RPM for 5 min and the pellets were re-suspended in 100 μl binding buffer containing 1 μl of Alexa Fluor 700 labelled anti-Annexin V secondary antibody. After 15 min, we washed the samples once with PBS and re-suspended the pellets in 300 ml PBS. We then used cells for FACS analysis (Guava) and analysed the generated data using FlowJo 8.6.6 Software.

#### Propidium Iodide staining and FACS analysis

We dissolved Propidium Iodide in distilled water, aliquoted the stock solution, and then stored these at −20 °C until use. For FACS analysis, Ln229 and MDA-MB231 cells were plated in 6-well plates at 2 × 10^5^ cells/well in DMEM medium supplemented with 10% FBS. We treated cells with different concentrations of drugs for various durations as described above and used for FACS analysis. After treatment, we collected the cells by trypsinization, re-suspended them in 0.5 ml ice cold PBS, and then fixed by adding 2 ml of 70% ice cold ethanol. We stored the samples at −20 °C until FACS analysis. Briefly, cells were spun, washed once in PBS, and re-suspended in 0.5 ml PBS/RNase A (10 µg/ml)/Triton X-100 (0.1%) buffer containing 0.5 µg/ml of Propidium Iodide (PI). After 15 min of incubation at room temperature in the dark, we washed cells once with PBS and FACS analysed (Guava) them. We analysed the generated data using FlowJo 8.6.6 Software for live and dead cells, and for cell cycle status analysis.

#### Caspase 3/7 assay

We performed a Caspase 3/7 assay by following the protocol provided by the manufacturer (Promega, Madison, WI).

## Electronic supplementary material

Supplementary materials
