# Supplementary material for: Comparison of cell-based assays to quantify treatment effects of anticancer drugs identifies a new application for Bodipy-L-cystine to measure apoptosis
Source: Sci Rep. 2018 Nov 5;8:16363. doi: 10.1038/s41598-018-34696-x (PMC6218539; doi:10.1038/s41598-018-34696-x)
Supplement: Supplementary file 1 — Supplementary materials [file 41598_2018_34696_MOESM1_ESM.pdf]

Supplementary Materials

Comparison of cell-based assays to quantify treatment effects of anticancer drugs identifies a new application for Bodipy-L-cystine to measure apoptosis

Nita Kumar[1], Rayhaneh Afjei[2], Tarik F. Massoud[2], and Ramasamy Paulmurugan[1]*

[1]Cellular Pathway Imaging Laboratory (CPIL), Molecular Imaging Program at Stanford, Stanford University School of Medicine, 3155 Porter Drive, Palo Alto, CA 94305, USA.
and

[2]Laboratory of Experimental and Molecular Neuroimaging (LEMNI); Molecular Imaging Program at Stanford; Stanford University School of Medicine, 300 Pasteur Drive,
Grant S-031, Stanford, CA 94305, USA.

Supplementary Figures

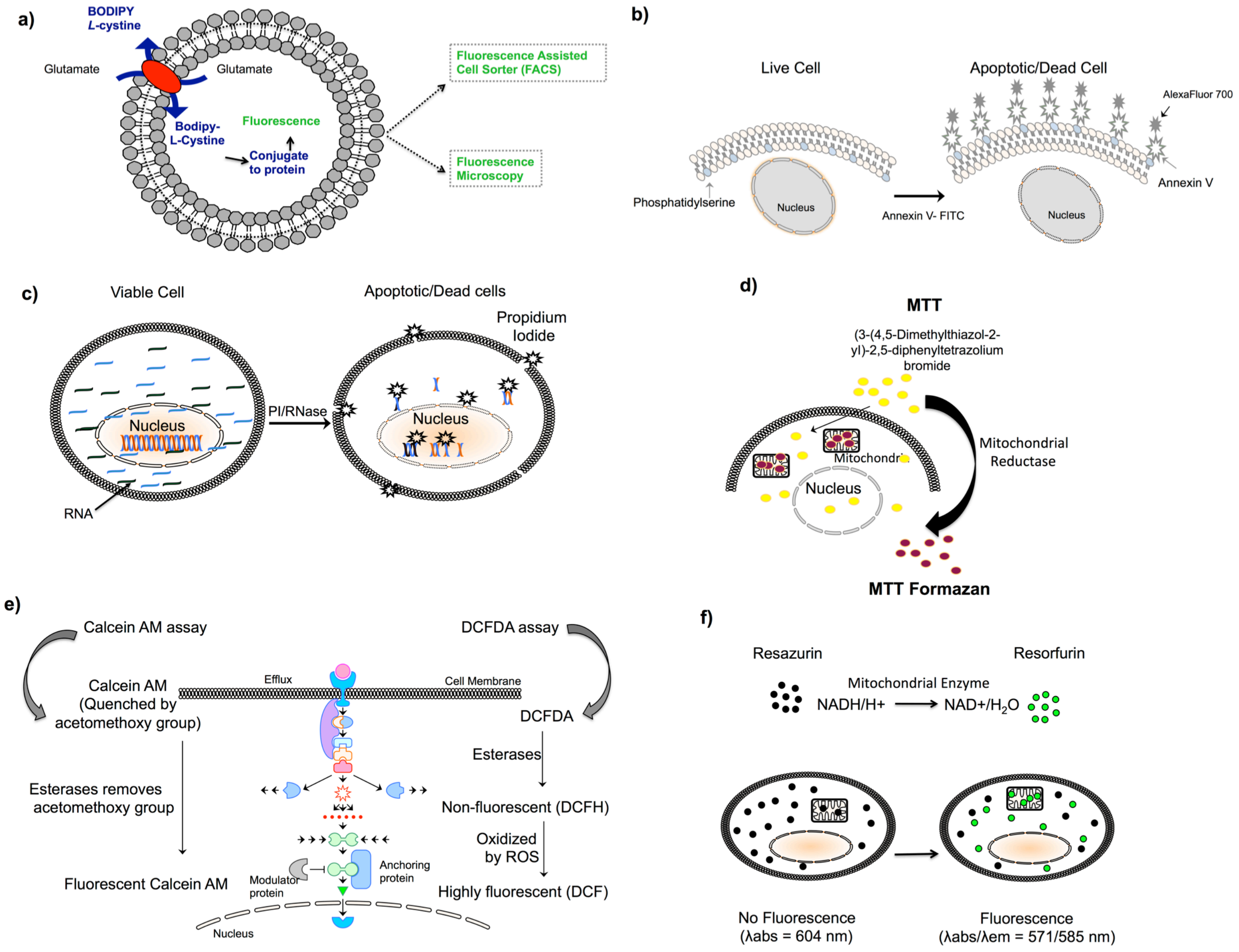

Supplementary Figure 1. Schematic illustration of the principles of different assays targeting cellular mechanism to measure cell viability and apoptosis. **a)** Schematic illustration of cystine/glutamate by xCT-cystine/glutamate antiporter and the concept of BFC apoptosis imaging in cells. **b)** Schematic illustration of the inversion of phosphatidylserine to the cells surface in which Anexin V dye binds to and fluoresces**. c)** Propidium Iodide is a nuclear dye that binds to double stranded DNA by intercalating through base pairs in cells that are fixed (permeabilized). Measuring the localized high intensity PI-fluorescence is an indicator of live cells while diffuse low-intensity fluorescence measures the apoptotic cells (DNA fragmentation). Propidium Iodide based

FACS analysis indirectly measure the cellular DNA contents and its distribution, which shows some advantage in measuring both cell cycle distribution and apoptosis. **d)** MTT assay is a colorimetric assay that asses cell viability based on the ability of viable cells to reduce the tetrazolium salt into a formazan product in the mitochondria. **e)** Calcein AM is a dye that measures the ROS (reactive oxygen species) within a cell. Once the dye diffuses into the cell, it is deacetylated by esterases to a non-fluorescent compound which is then oxidized by ROS into a highly fluorescent compound. Calcein AM is a non-fluorescent dye that can readily enter viable cells. Once inside the cells, the AM (acetomethoxy) is cleaved by esterases and the fluorescent calcein portion remains. Only viable cells can be labelled with calcein since the esterase activity is only present in viable cells. **(e-right)**. The DCFDA assay is an assay that measures the ROS within a cell. Once the dye diffuses into the cell, it is deacetylated by intracellular esterases to a non-fluorescent compound (H_2DCFDA-AM), which is then oxidized by cellular ROS into a highly fluorescent compound (2'-7'dichlorofluorescein (DCF)) **(e- left)**. **f)** Cell Titer Blue is an assay that is based on a cell's ability to convert a redox dye (resazurin) into a fluorescent end product (resofurin).

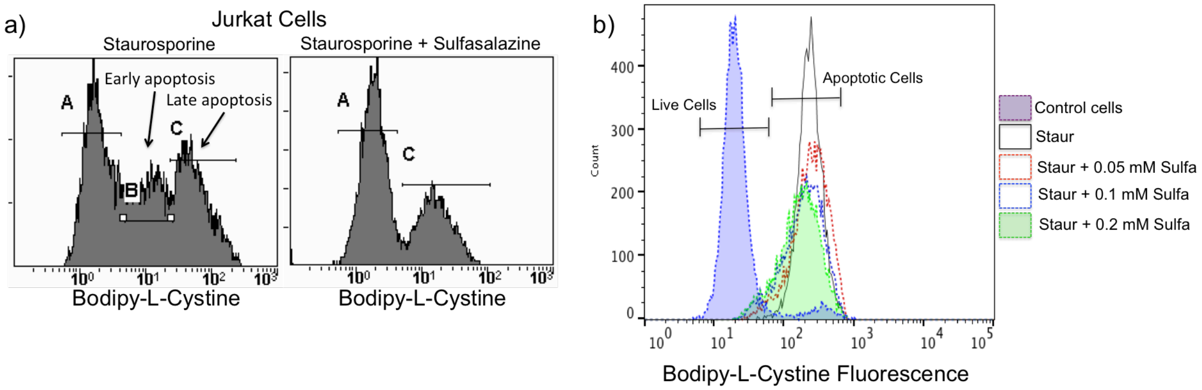

Supplementary Figure 2. To further confirm the uptake of BFC is occurring via xCT-cystine/glutamate antiporter, we used sulfasalazine, an inhibitor of glutamate/cystine antiporter. The jurkat cells treated with staurosporine (500 nM) were co-incubated with 1 nM of BFC and 0.15 mM of sulfasalazine for 30 mins at 37°C and analysed by FACS. The results showed significant reduction in the fluorescent signal from apoptotic cells when co-treated with sulfasalazine **(a)**. Similarly, MDA MB231 cells treated with 100 nM staurosporine and assessed for the sulfasalazine dose (0.05, 0.1 and 0.2 mM) dependent inhibition of BFC uptake. The results showed significant sulfasalazine dose

dependent reduction in the fluorescent signal from apoptotic cells when co-treated with sulfasalazine **(b)**.

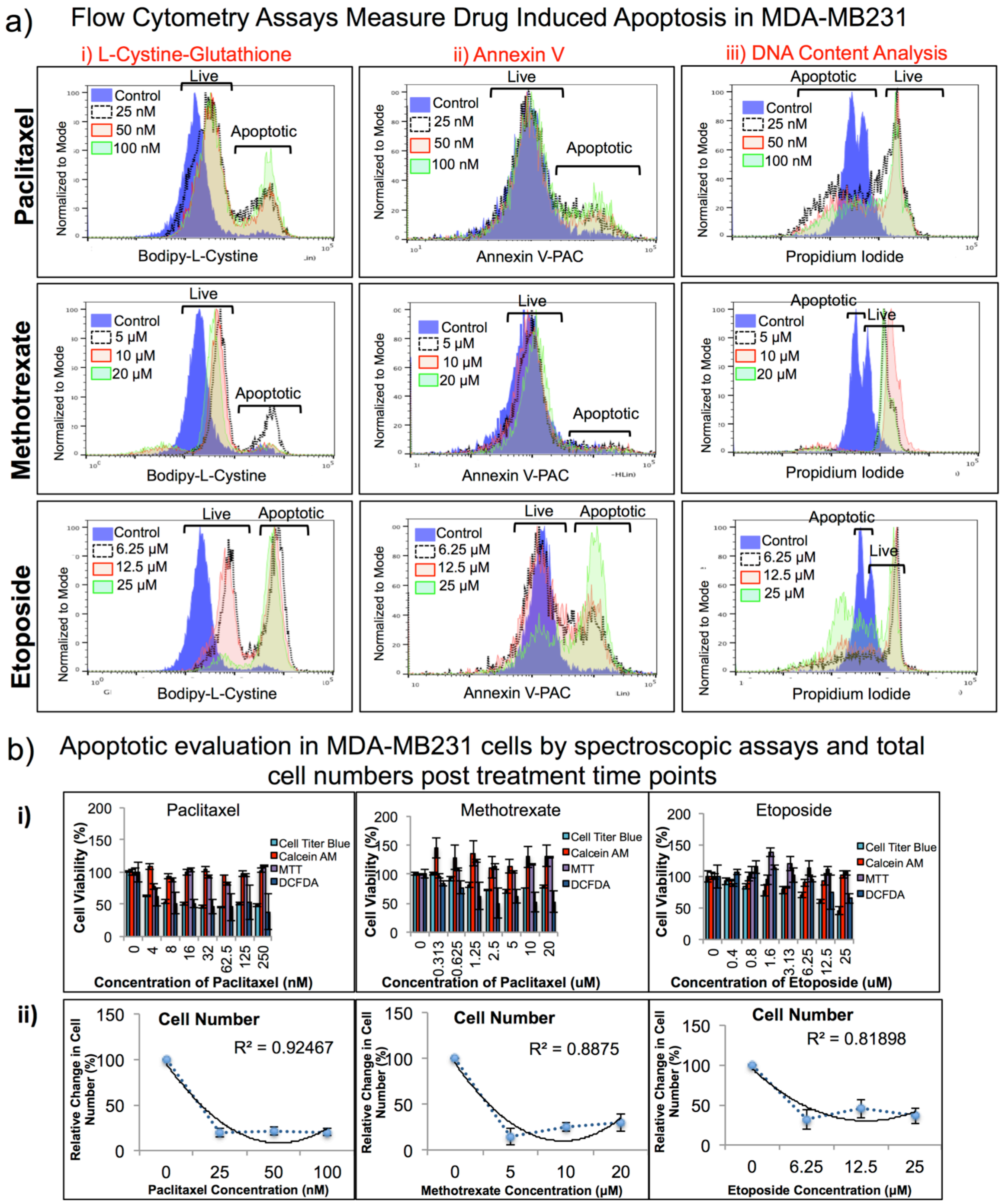

Supplementary Figure 3. a) Flow cytometry assays measure drug-induced apoptosis in MDA-MB231 cells. Comparison of anticancer drugs in MDA-MB231 cells

was evaluated by three different commonly used FACS assays (PI staining based DNA content analysis, Annexin V based staining, and BFC based apoptosis staining) to cover cell viability, cell proliferation, cellular stress, apoptotic and live cell populations, and total number of cells present after 48 h post drug treatment. The results also found that not all assays measure the cell viability at equal sensitivity. **b) Spectroscopy assays measure drug-induced apoptosis in MDA-MB231.** We used four different spectroscopy assays (MTT, Calcein-AM, Cell Titer Blue, and DCFDA). The results also found that not all assays measure the cell viability at equal sensitivity. The Cell Titer Blue assay worked well with higher rate of consistency compared to other spectroscopic assays. We also measured total cell numbers 48 h after drug treatment.

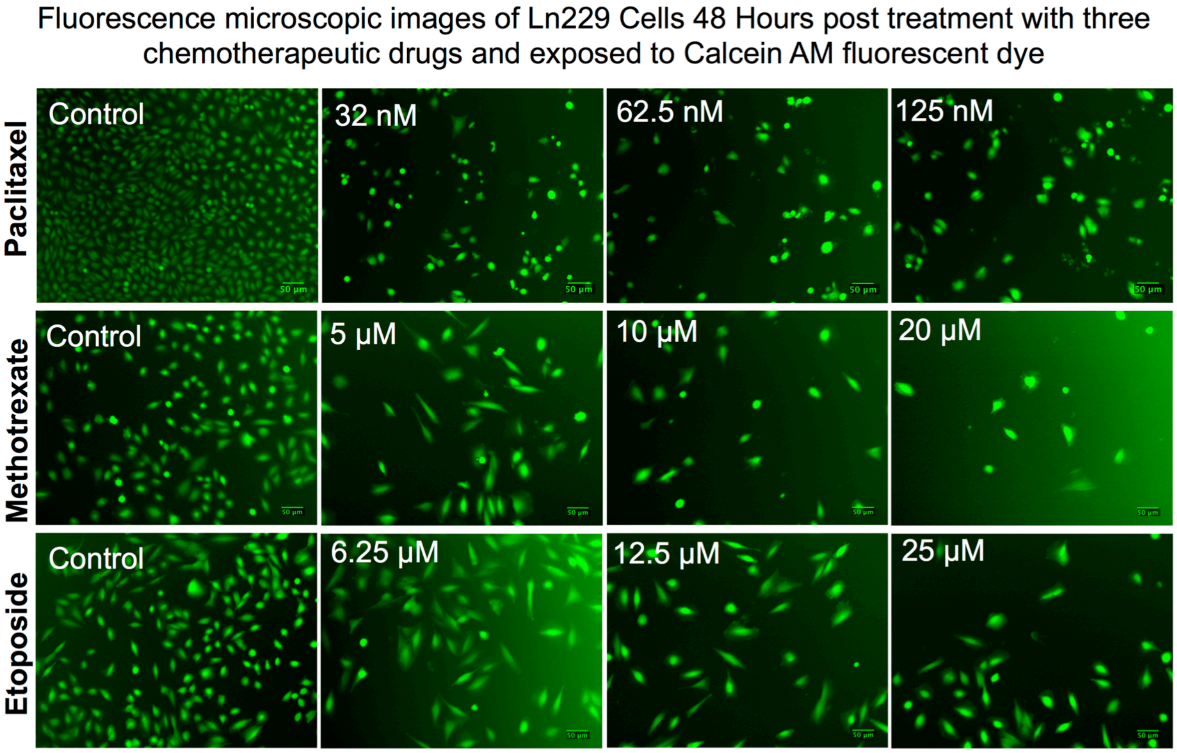

Supplementary Figure 4. Fluorescence microscopic images of Ln229 Cells 48 h post treatment with three different chemotherapeutic drugs, and stained with Calcein AM fluorescent dye. Fluorescent microscopy images of Ln229 cells stained with Calcein AM (green; live cells) for live and dead cell differentiation after apoptosis induction with paclitaxel (top), methotrexate (middle), and etoposide (bottom) taken after 48 h of drug treatment.

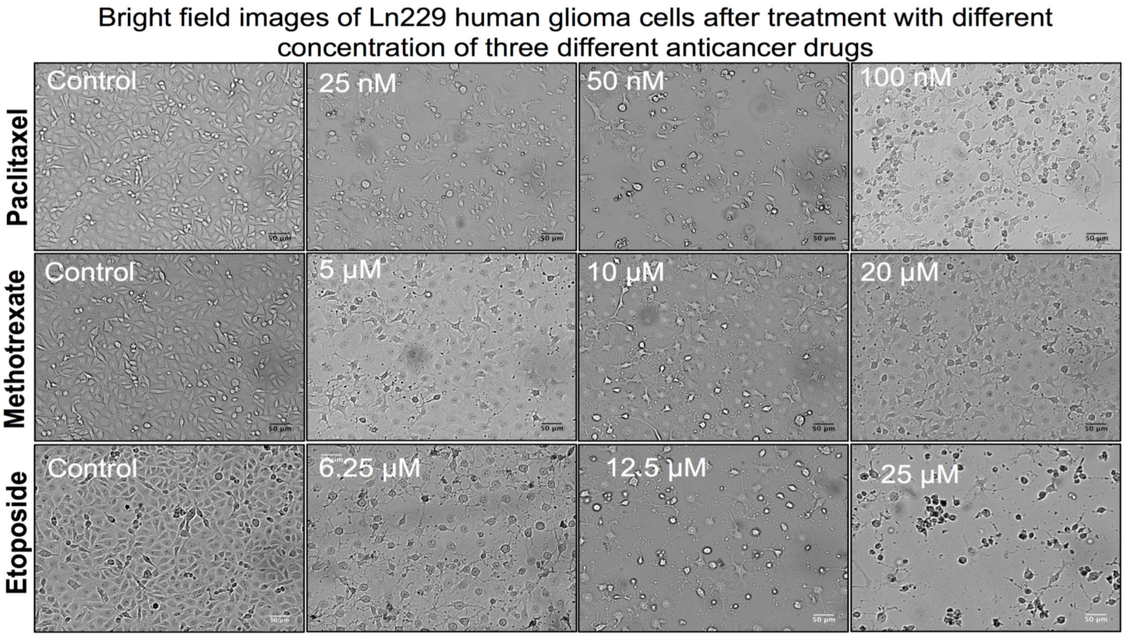

Supplementary Figure 5. Brightfield microscopy images of MDA-MB231 cells treated with paclitaxel (top), methotrexate (middle) and etoposide (bottom) taken 48 h after drug treatment.

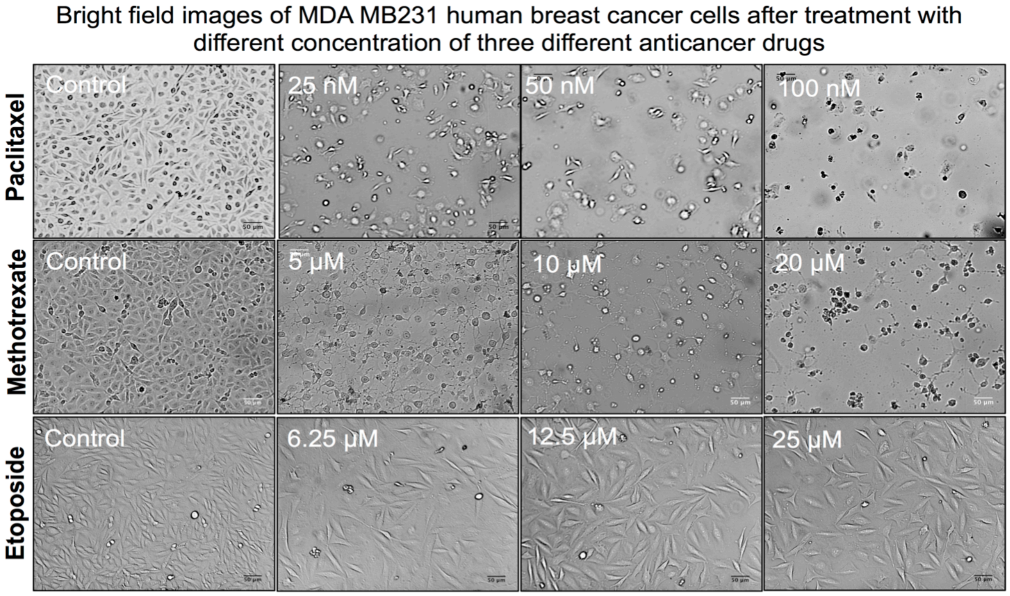

Supplementary Figure 6. Brightfield microscopy images of Ln229 cells treated with paclitaxel (top), methotrexate (middle), and etoposide (bottom) taken 48 h after drug treatment.

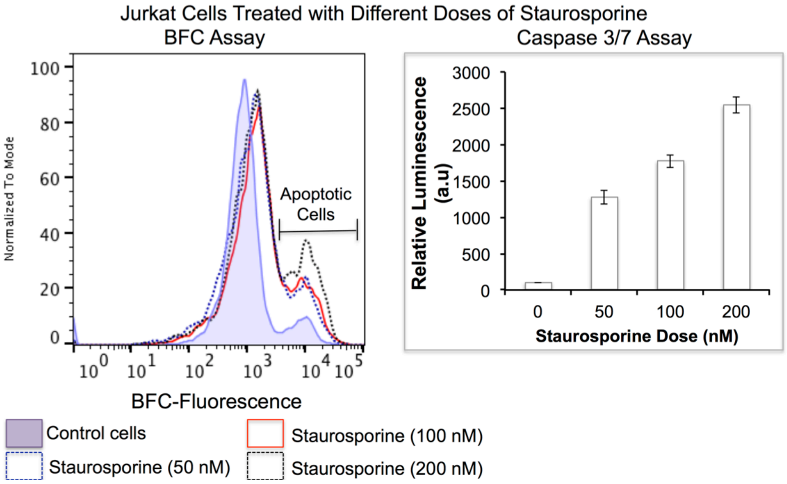

Supplementary Figure 7. Jurkat cells treated with different concentration of staurosporine (0, 50, 100 and 200 nM) for 6 h were simultaneously assessed for BFC uptake by FACS assay (left) and Caspase 3/7 activity by Promega Luciferase-Luciferin based DEVD cleavage assay (right).

Supplementary Tables:

Supplementary Table 1: Apoptotic cells population measured at different treatment conditions by various assays:

Ln229 Cells	Paclitaxel			
	0	25nm	50nm	100nm
Bodipy-L-Cystine	9.46	30.9	55.6	66.7
Annexin V	1.44	17.9	46.3	57.1
PI	0.062	3.96	49.5	54.5

Ln229 Cells	Methotrexate			
	0	5µm	10µm	20µm
Bodipy-L-Cystine	6.07	15.2	16.5	19.3
Annexin V	1.27	2.61	3.23	2.69
PI	0.17	0.43	0.36	0.47

Ln229 Cells	Etoposide			
Assays	0	6.25µm	12.5µm	25µm
Bodipy-L-Cystine	6.6	18.2	21	18.1
Annexin V	0.57	2.9	1.52	2.46
PI	0.11	1.40	1.32	0.47

Supplementary Table 1: Apoptotic cells populations measured at different treatment conditions by various assays from Figure 3a.

Supplementary Table 2: Flow cytometry assays measure paclitaxel induced apoptosis in MDA MB231 cells at different time points post treatment:

**Assays**	**Paclitaxel -6 hrs**			
	0	25nm	50nm	100nm
**Bodipy-L-Cystine**	7.35	6.72	49.4	48.7
**Annexin V**	3.94	1.93	2.76	3.36
**PI**	0.91	0.68	1.16	1.44
**Assays**	**Paclitaxel -12 hrs**			
	0	25nm	50nm	100nm
**Bodipy-L-Cysteine**	5.99	7.47	7.39	9.35
**Annexin V**	13.2	22	29.4	18
**PI**	1.36	6.47	3.47	3.81
**Assays**	**Paclitaxel -24 hrs**			
	0	25nm	50nm	100nm
**Bodipy-L-Cysteine**	9.04	13.9	8.96	8.82
**Annexin V**	4.55	6.14	5.56	8.20
**PI**	1.61	22.6	17.4	10.4

Supplementary Table 2: Flow-cytometry assays measure paclitaxel-induced apoptosis in MDA MB231 cells at different time-points post treatment from Figure 5a.

Supplementary Table 3. Apoptotic cell populations measured at various drug treatment conditions by different assays:

MDA MB231 Cells	Paclitaxel			
	0	25nm	50nm	100nm
Bodipy-L-Cystine	5.84	24	23.4	34
Annexin V	0.023	33.3	29.9	13.5
PI	0.71	31.3	45.7	35.8

MDA MB231 Cells	Methotrexate			
	0	5µm	10µm	20µm
Bodipy-L-Cystine	5.25	24.3	6.9	8.59
Annexin V	0.21	9.58	7.40	6.05
PI	1.38	3.67	5.35	3.68

MDA MB231 Cells	Etoposide			
	0	6.25µm	12.5µm	25µm
Bodipy-L-Cystine	4.80	54.3	50.1	85
Annexin V	5.37	51.3	62.1	64.7
PI	0.23	27	36.1	61.7

Supplementary Table 3: Apoptotic cells populations measured at different treatment conditions by various assays from Supplementary Figure 3a.
